# The relationship between psychological resilience and professional quality of life among mental health nurses: a cross-sectional study

**DOI:** 10.1186/s12912-023-01346-1

**Published:** 2023-05-29

**Authors:** Ohoud Alonazi, Amira Alshowkan, Emad Shdaifat

**Affiliations:** 1 Master of Psychiatric and Mental Health Nursing College of Nursing, Imam Abdulrahman Bin Faisal University, Dammam, Saudi Arabia; 2 Community Nursing Department, College of Nursing, Imam Abdulrahman Bin Faisal University, P.O. Box 1982, Dammam, Saudi Arabia

**Keywords:** Psychological resilience, Empathy, Burnout, Secondary traumatic stress, Nurses

## Abstract

**Background:**

Mental health nursing is a demanding and stressful profession that impacts nurses’ professional quality of life. Psychological resilience can be a protective factor. However, the relationship has not been extensively studied. This study aims to examine the relationship between psychological resilience and professional quality of life and identify potential predictors of ProQOL subscales among mental health nurses.

**Methods:**

The study employed a cross-sectional design to collect data from 179 mental health nurses. Data was collected using two standardized questionnaires: the Connor-Davidson resilience scale and the professional quality of life scale. Participants were recruited through convenient sampling during a 3-month period from April to June 2022, and the data were collected using an online survey tool called QuestionPro.

**Results:**

The study found a strong positive correlation between psychological resilience and compassion satisfaction (*r* = 0.632, *P* < 0.001). However, there was a negative significant correlation between resilience with burnout (*r* = -0.470, *P* < 0.001) and secondary traumatic stress (*r* = -0.210, *P* = 0.005). The study also found that higher resilience levels were associated with higher levels of compassion satisfaction and lower levels of secondary traumatic stress. Additionally, higher burnout scores were associated with higher levels of secondary traumatic stress. The study also identified that age and the number of children had weak associations with compassion satisfaction, while workplace was a significant predictor of burnout and secondary traumatic stress.

**Conclusion:**

The study emphasizes the importance of resilience, burnout, and secondary traumatic stress in the well-being of healthcare professionals, especially nurses. The findings suggest that assessing nurses’ resilience and professional quality of life can raise psychological resilience awareness and help managers create the necessary working conditions to improve nurses’ professional quality of life.

## Introduction

Mental health nurses are at a greater risk of being subjected to stress as they work in a demanding environment and have direct interaction with psychiatric patients [1]. They encounter challenging situations, including patient seclusion, attempted suicides, physical and verbal assaults [2–4], and have to limit patients’ behavior, which can lead to feelings of guilt and fear [5]. Patients’ aggression and violence can also invade nurses’ safe workplace, causing emotional distress in carrying out their job [6, 7]. This stress can cause physical and mental health problems, such as fatigue, tedium, and burnout [8]. The detrimental effect on the professional quality of life (ProQOL) of mental health nurses caused by organizational factors, such as an increase in workload and a shortage of resources, has been well established in the literature [9, 10].

The cumulative effects of occupational stressors and challenges for mental health nurses can lead to adverse effects, including long-term stress, emotional exhaustion, and may even lead to post-traumatic stress disorder and depression [6, 11, 12]. Job dissatisfaction among nurses is related to workplace stress [13] and can adversely affect job retention [14]. Frequent exposure to traumatic conditions from patients may also reduce the quality of nurses’ careers and lead to negative patient outcomes [15]. Hence, examining the ProQOL among mental health nurses is essential in identifying proactive measures to alleviate the adverse effects of workplace stress.

According to the Mental Health Atlas 2020, there were only 872 mental health nurses in Saudi Arabia, with a ratio of 2.54 nurses per one hundred thousand population. In comparison, the number of psychiatrists was 1170, with a ratio of 3.41 per one hundred thousand population, and the number of social workers was 2909, with a ratio of 8.49. This indicates that the number of mental health nurses in Saudi Arabia is relatively low, which may result in work overload and increase the risk of burnout [16]. Hence, exploring factors linked to nursing resilience and ProQOL of life in mental health nurses in Saudi Arabia is imperative. Such research can help identify potential strategies to mitigate the adverse impact of work-related stress.

The concept of resilience has gained global attention as a strategy to alleviate the adverse effects of job-related stressors and to prevent various psychosocial problems among nurses [17, 18]. Psychological resilience refers to the nursing staff’s ability to adapt to workplace stress [8]. The theoretical model of workplace resilience aligns individual characteristics with resilience that may influence psychological functioning. Resilience is considered a critical factor that greatly impacts an individual’s subsequent psychological function, and includes variables such as neuroticism, vigilance, self-efficacy, and coping [19]. For instance, studies have consistently shown that high levels of neuroticism are associated with negative psychological distress, leading to high levels of depression and anxiety [20–22]. Furthermore, mental health nurses’ job performance has been linked to their mental health [23]. Studies have indicated a positive relationship between the resilience of mental health nurses and their job satisfaction [24] and life satisfaction [25]. It is crucial to recognize that organizations and employers, as well as individuals, share responsibility for building psychological resilience in the workplace.

Professional quality of life refers to an individual’s satisfaction and perception of their workplace, job effectiveness, and productivity. It encompasses work-related pleasure and the ability to cope with work-related stressful circumstances. ProQOL has a significant impact on an individual’s overall work satisfaction [26]. Compassion satisfaction (CS) is the feeling of achievement resulting from supporting and caring for others [27]. A high level of CS provides the benefit of allowing nurses to provide quality and effective nursing care, as well as making them more optimistic and compassionate in their work. Some variables like age, gender, marital status, and working shift were linked to CS. Precisely, working in shifts, in primary care facilities and urban areas was related to reduction of CS. Nevertheless, CS was found high among the divorced profession, and burnout was related only to being working in shifts [28]. Some studies reported that nurses are at high risk for burnout (BO) and secondary traumatic stress (STS) in comparison to other healthcare staff due to long working hours and workload challenges [29, 30].

Healthcare professionals face unique challenges in their work environments that can have negative impacts on their well-being and job performance. Resilience, defined as the ability to adapt and cope with adversity, has been identified as a critical factor in helping healthcare professionals maintain their well-being and job satisfaction in the face of these challenges [31]. Additionally, professional quality of life, which includes dimensions of compassion satisfaction, burnout, and secondary traumatic stress, has been shown to affect healthcare professionals’ job performance and patient care [32]. Previous research has identified strong relationships between resilience, professional quality of life, and job satisfaction in healthcare settings, highlighting the importance of understanding these concepts and their interactions in promoting the well-being and effectiveness of healthcare professionals [33, 34].

In Saudi Arabia, a study found a significant relationship between BO and employment location, nursing department, and age. Both employment location and nursing department had a significant influence on STS, and Saudi nurses had high levels of CS and moderate levels of BO and STS [35]. Another study among primary healthcare nurses found high levels of BO linked with job stressors, age, educational level, and sources of workplace stress [36]. Among mental health nurses in Turkey, a positive link was discovered between nurses’ professional values, compassion, fulfillment, and BO. Professional values, education level, and time spent on social activities were significant predictors of ProQOL fatigue [37]. Another study reported a link between CS and BO, as well as psychological resilience and BO and compassion fatigue (CF) [18]. Mental health nurses in South Africa were found to have higher levels of BO, STS, and lower CS compared to other nurse professions [38]. In Greece, most mental health nurses reported experiencing low CS, nearly half of them experienced increased BO, and almost half had a high risk for STS [39]. Therefore, regular assessment of nurses’ resilience, BO, and ProQOL is recommended, along with the use of educational programs to boost nurses’ resilience and CS while reducing BO.

This study aimed to investigate the relationship between psychological resilience and ProQOL among mental health nurses, which has not been extensively researched. The demanding and stressful nature of the mental health nursing profession makes it crucial to explore factors that impact nurses’ well-being. The study’s novelty lies in its examination of potential predictors of the Professional Quality of Life subscales, which can inform interventions and support systems to improve mental health nurses’ well-being and job satisfaction.

The study aims to fill the knowledge gap by examining the relationship between psychological resilience and ProQOL among mental health nurses in the Eastern Region of Saudi Arabia. It seeks to determine the level of psychological resilience and ProQOL domains, as well as the correlation between them. Furthermore, the study aims to identify the predictors of different subscales of ProQOL. The main hypothesis is that there is a positive relationship between psychological resilience and the ProQOL of mental health nurses. To achieve the objectives, the study will explore the following research questions:

- What is the level of psychological resilience among mental health nurses?
- What are the levels of the ProQOL domains among mental health nurses?
- Is there a correlation between psychological resilience and ProQOL among mental health nurses?
- What factors predict the different subscales of ProQOL?

## Methods

### Setting and design

Data was collected from mental health nurses working at the Mental Health Center in Riyadh, Saudi Arabia using a cross-sectional design. This government-run center provides free psychiatric, psychological, and social care to patients with mental health disorders, and houses 665 patient beds. The center serves all 12 regions of Saudi Arabia, and is operated by the Ministry of Health, with the largest center located in Riyadh. The main distinguishing factors between centers are the number of staff and workload.

### Sampling and sample size

The total population of interest in the study was identified as eligible nurses who provided direct care to patients with mental illness in in-patient units, out-patient units, or emergency rooms, and had worked for at least 3 months. The total population was determined to be 320. The researchers used Slovin’s formula to determine the minimum required sample size. An acceptable margin of error or level of precision of 5% was chosen for the study. The formula used was *n* = N / (1 + Ne^2), where n represented the sample size, N was the total population, and e was the margin of error. The values were substituted into the formula, and *n* = 320 / (1 + (320 × 0.05^2)) was obtained. The formula was then simplified to *n* = 320 / (1 + 0.8). Solving for n, *n* = 320 / 1.8. The calculated sample size was 177.78, which was rounded up to the nearest whole number since fractional sample sizes were not possible. Therefore, the minimum required sample size for the study was determined to be 178 nurses. The data was collected from nurses using a convenience sampling technique between April and June 2022. The researchers were present at the sampling locations during data collection to address any inquiries that the participants might have had and to clarify the aim of the study.

**Fig. 1:**
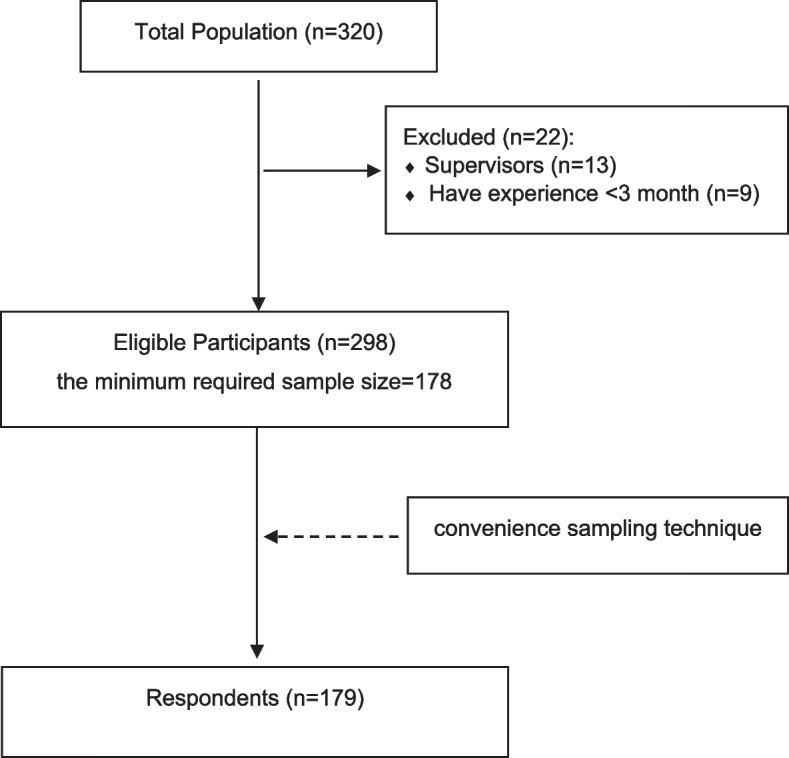
Flow diagram of participant recruitment and selection

The study included all nurses who directly cared for patients with mental illness in the in-patient psychiatric unit, out-patient unit, or emergency room for more than 3 months. Exclusion criteria were supervisors and nurses with limited experience (Fig. 1). The purpose was to obtain a homogeneous sample population with similar levels of exposure to mental health care challenges. The criteria were written clearly and concisely for easy understanding by potential participants and to ensure consistency and relevance to the study objectives.

### Instruments

Two tools were used in this study to collect data. The first tool was the Connor-Davidson Resilience Scale (CD-RISC-25), which measures resilience through 25 items scored on a five-point scale ranging from 0 to 4. The CD-RISC-25 is a questionnaire that assesses various aspects of resilience. It includes statements that measure different components of resilience, such as hardiness (including commitment, challenge, and control), coping mechanisms, adaptability and flexibility, meaningfulness and purpose, optimism, regulation of emotion and cognition, and self-efficacy. The scale was used to evaluate participants’ resilience levels based on their experiences in the past month. The total score ranges from 0 to 100, with higher scores indicating higher levels of resilience. The CD-RISC-25 has been tested for reliability and validity in various contexts and has been modified into different versions. Examples of items on the scale include “I am able to adapt when changes occur”, “I can deal with whatever comes my way”, and “I tend to bounce back after illness, injury, or other hardships”. In previous research, the scale has demonstrated high reliability, with a coefficient alpha of 0.89 [40].

Furthermore, the Arabic version of the CD-RISC has demonstrated high reliability with a Cronbach’s alpha coefficient of 0.89, indicating its validity and reliability in evaluating resilience among Arabic-speaking individuals [41]. Additionally, the CD-RISC has been found to possess favorable psychometric properties with a Cronbach’s alpha coefficient of 0.89 [42], while previous research has reported a strong internal consistency of the scale with a coefficient alpha value of 0.79 [43]. In our study, the internal consistency of the CD-RISC was 92.8% as measured by Cronbach’s alpha.

Secondly, the ProQoL is a tool developed by Stamm et al., (2005) to measure the positive and negative aspects that impact the quality of professional life for helping professionals. The scale used in the study is composed of 30 tripartite items that cover the three dimensions identified above: Compassion Satisfaction (10 items), Burnout (10 items), and Secondary Traumatic Stress (10 items). Respondents are asked to rate their experiences over the past 30 days using a 6-point Likert scale ranging from 0 (“never”) to 5 (“very often”). The instrument used in the study includes various items that assess different aspects of psychological well-being. For example, to measure Compassion Satisfaction, items such as “I have positive thoughts and feelings about those I help and how I could help them” were included. Conversely, items such as “I feel overwhelmed by the amount of work or the number of clients I have to deal with” were included to measure Burnout, and “I am jumpy and easily startled” was included to measure Secondary Traumatic Stress. Some items were reverse-coded, such as 1, 4, 15, 17, and 29. Compassion Satisfaction, Burnout, and Secondary Traumatic Stress were calculated by summing the values assigned to specific items.

The ProQOL scale used in this study consists of three subscales: Compassion Satisfaction (CS), Burnout (BO), and Secondary Traumatic Stress (STS). Each subscale has a reliability coefficient value of 0.87, 0.72, and 0.81, respectively. Higher scores on the subscales indicate higher levels of CS, BO, or STS. The total score can be used to determine the level of ProQOL and is classified as low (≤ 22), moderate (23–41), or high (≥ 42). The ProQOL scale has been used for more than 20 years and in over 200 studies [32]. The Arabic version of the ProQOL yielded Cronbach’s alpha values of 0.84, 0.78, and 0.73 for compassion satisfaction, secondary traumatic stress, and burnout, respectively [44]. In a separate study conducted in Saudi Arabia, the ProQOL subscales demonstrated good internal consistency, with Cronbach’s alpha values of 0.91, 0.76, and 0.82 for the CS, BO, and STS subscales, respectively [35]. In our study, we found the reliability alpha to be 88.3%, 72.1%, and 80.5% for the CS, BO, and STS subscales, respectively.

To establish validity, the findings were compared to the critical value table and the total value was matched with the results. Construct validity of each question was assessed using Pearson correlation analysis. A question was considered valid if the r value was above 0.1467, as determined by a table of Pearson correlation analysis. Resilience items had a range of 0.713 to 0.386, while ProQOL subscales were as follows: CS (0.781–0.651), BO (0.733–0.261), and STS (0.760–0.270). These results confirm the construct validity of the questionnaire.

### Ethical considerations

Prior to commencing the study, ethical approval was sought and granted from the Institutional Review Board (IRB) at Imam Abdulrahman bin Faisal University. Additionally, approval was obtained from the Eradah complex and Mental Health in Riyadh. The participants were given a thorough explanation of the study’s objectives, benefits, confidentiality, and their voluntary participation before they gave their informed consent to participate. The study was conducted in compliance with the ethical principles outlined in the Declaration of Helsinki, and steps were taken to ensure the privacy and confidentiality of the participants. All data collected was treated in accordance with ethical guidelines. The researchers identified and minimized any potential risks to the participants. The study strictly adhered to the highest ethical standards for research that involves human subjects.

### Data collection

Once we received the consent letter from Eradah Complex and Mental Health in Riyadh, we developed an online survey using QuestionPro, a survey technology that enables simple distribution and response gathering. The survey was designed to gather data on the perspectives and experiences of nurses working in mental health center in Riyadh and employed Likert scale questions. We then made contact with the nurses who were on duty at the mental health facilities via email or other electronic communication methods and provided them with a link to the survey. Participants were informed that their participation was voluntary and that their responses would remain anonymous. To ensure a high response rate, we reminded participants daily to complete the survey and made ourselves available to answer any questions they may have had about the survey or the research project as a whole.

### Data analysis

The statistical analyses were performed using SPSS for Windows version 20.0 (SPSS, Chicago, IL). Continuous data were presented as mean and standard deviation (SD), while categorical data were presented as numbers and percentages. Correlations between resilience and ProQOL were tested using a correlation coefficient test, and multiple linear regression was used to identify independent predictors of ProQOL subscales. Demographic variables such as gender, marital status, having children, education level, and workplace were included as dummy variables. Three cases with missing data for key variables were excluded, and variables with randomly missing data were imputed using the marginal median. Statistical significance was set at *p* < 0.05. These analyses were conducted to determine the relationship between resilience and ProQOL, and to identify factors that may predict ProQOL subscales.

## Results

A total of 179 mental health nurses participated in this study. The average age of the participants was 33.8 years, with a standard deviation of 6.7. The majority of the mental health nurses were married (64.8%) and held a Bachelor of Science in Nursing degree (55.3%). Additionally, almost two-thirds of the respondents reported having children. The average years of experience in mental health nursing was 11.3 years, with a standard deviation of 6.8. In terms of work setting, half of the mental health nurses worked in inpatient wards, while 35.8% of them worked in outpatient departments (Table 1).

**Table 1:** Sociodemographic characteristics of mental health nurses (*N* = 179)

	N	%
**Age (years)**		
<30	49	27.4
30–40	108	60.3
>40	22	12.3
**Mean ± SD**	33.8 ± 6.7	
**Gender**		
Male	90	50.3
Female	89	49.7
**Marital Status**		
Single	47	26.3
Married	116	64.8
Divorced/Widowed	16	8.9
**Having children**		
Yes	107	59.8
No	72	40.2
**How many children**		
<3	46	43.0
3–5	44	41.1
>5	17	15.9
**Educational Level**		
Diploma	55	30.7
Technical institute of nursing	10	5.6
Bachelor of Science in Nursing	99	55.3
Postgraduate	15	8.4
**Experience Years**		
<10	78	43.6
10–20	84	46.9
>20	17	9.5
**Mean ± SD**	11.3 ± 6.8	
**Workplace**		
In-patient	92	51.4
Out-patient	64	35.8
Emergency room	23	12.8

The results of the study showed that the participants had a high mean score of total psychological resilience, with a mean of 94.6 and a standard deviation of 15.7. When examining the specific domains of resilience, the mean score of hardiness was 27.4 with a standard deviation of 5.2, indicating that the participants had confidence in dealing with new challenges and believed they could achieve their goals, even in the presence of obstacles. The mean score of coping was 18.2 with a standard deviation of 3.6, indicating that the participants reported having a close and secure relationship, and felt capable of coping during times of stress or crisis (Table 2).

**Table 2:** Mean and SD psychological resilience domains scores of mental health nurses (*N* = 179)

	Mean ± SD
Hardiness	27.4 ± 5.2
Coping	18.2 ± 3.6
Adaptability/flexibility	11.4 ± 2.4
Meaningfulness/purpose	15.3 ± 2.8
Optimism	6.9 ± 1.8
Regulation of emotion and cognition	6.9 ± 1.9
Self-efficacy	8.2 ± 1.5
**Total Resilience Score**	94.6 ± 15.7

The results of the study indicated that around two-thirds (58.1%) of the mental health nurses had good psychological resilience. When examining the specific domains of the psychological resilience scale, approximately two-thirds of the participants had good levels of hardiness (63.7%), meaningfulness/purpose (61.5%), and self-efficacy (66.5%). In terms of coping, half of the studied mental health nurses (50.3%) had good scores in this domain (Table 3).

**Table 3:** Frequency distribution of psychological resilience domains scores of the mental health nurses (*N* = 179)

	Poor		Average		Good	
	N	%	N	%	N	%
Hardiness	16	8.9	49	27.4	114	63.7
Coping	33	18.4	56	31.3	90	50.3
Adaptability/flexibility	20	11.2	73	40.8	86	48.0
Meaningfulness/purpose	8	4.5	61	34.1	110	61.5
Optimism	38	21.2	72	40.2	69	38.5
Regulation of emotion and cognition	44	24.6	60	33.5	75	41.9
Self-efficacy	4	2.2	56	31.3	119	66.5
**Total Resilience Score**	19	10.6	56	31.3	104	58.1

More than half (57%) of the mental health nurses had an average level of compassion satisfaction domain, indicating that they feel fulfilled and satisfied in their work with patients. Only 1.7% of them had a low level of compassion satisfaction, indicating a need for further support and interventions to improve their job satisfaction. In terms of burnout domain, three-quarters of the nurses (70.9%) had an average level, indicating moderate levels of emotional exhaustion and depersonalization. Regarding secondary trauma stress domain, about two-thirds of them (68.7%) had an average level, indicating that they experience moderate levels of stress and negative feelings related to their work with traumatized patients. However, only 4.5% had a good level of secondary trauma stress (Table 4).

**Table 4:** Frequency distribution of ProQOL scale domains scores of the mental health nurses (*N* = 179)

	Low		Average		Good	
	N	%	n	%	N	%
Compassion Satisfaction	3	1.7	102	57.0	74	41.3
Burnout	52	29.1	127	70.9	0	0.0
Secondary Traumatic Stress	48	26.8	123	68.7	8	4.5

Table 5 presents the mean scores and standard deviations for each dimension of ProQOL. The mean score for Compassion Satisfaction was 39.1 (SD 7.0), for Burnout was 26.1 (SD 5.9), and for Secondary Traumatic Stress was 27.8 (SD 7.3).

**Table 5:** The mean, standard deviation, minimum, maximum of ProQOL (*N* = 179)

	Min	Max	Mean	SD
Compassion Satisfaction	21.0	50.0	39.1	7.0
Burnout	10.0	41.0	26.1	5.9
Secondary Traumatic Stress	12.0	48.0	27.8	7.3

The results indicate that there is a statistically significant strong positive correlation between psychological resilience and the compassion satisfaction domain (*r* = 0.632, *p* < 0.001). On the other hand, there is a statistically significant negative correlation between psychological resilience and the burnout domain (*r* = -0.470, *p* < 0.001), as well as the secondary traumatic stress domain (*r* = -0.210, *p* = 0.005). These findings are summarized in Table 6.

**Table 6:** Correlation between psychological resilience and ProQOL domains level

	**Resilience**	
	***r***	***P***
**ProQOL**		
Compassion Satisfaction	0.632	< 0.001
Burnout	-0.470	< 0.001
Secondary Traumatic Stress	-0.210	0.005

The study utilized multiple regression analysis to investigate the impact of demographic variables and resilience levels on ProQOL subscales. The findings indicated that higher resilience levels were positively associated with compassion satisfaction (β = 0.499, *p* < 0.001), whereas the number of STS symptoms was negatively associated with compassion satisfaction (β = -0.274, *p* < 0.001). Additionally, higher BO scores were found to predict greater compassion satisfaction (β = 0.306, *p* < 0.001). The results also suggested that age and number of children had weak but positive and negative associations with compassion satisfaction (β = 0.152, *p* < 0.05), (β = 0.166, *p* < 0.05), respectively. Overall, the regression model demonstrated a good fit, as evidenced by the significant *F*-value (*F* = 11.877, *p* < 0.001), *R*-squared value (*R*^2^ = 0.51), and adjusted *R*-squared value (*R*^2^_adj = 0.467). Thus, the study showed that resilience, BO, STS, age, and number of children are significant predictors of compassion satisfaction in healthcare professionals (Table 7).

**Table 7:** Multiple regression analysis of predictors of ProQOL subscales

**Dependent Variables**	**Independent Variables**	***B***	***SE B***	***β***
**CS**	Constant	5.396	4.035	***-***
	BO	0.402	0.107	0.306**
	Resilience	0.223	0.028	0.499**
	STS	-0.269	0.078	-0.274**
	Gender (ref; Female)	-0.276	0.840	-0.020
	Education level (ref: BSc)			
	Diploma	0.190	0.935	0.012
	Technical institute of nursing	-1.464	1.773	-0.048
	Postgraduate	-2.732	1.539	-0.105
	Workplace (ref: Emergency)			
	In-patient	2.221	1.246	0.158
	Out-patient	1.866	1.371	0.126
	Marital Status (ref: Married)			
	Single	0.099	1.347	0.006
	Divorced/Widowed	1.748	1.483	0.072
	Having Children (ref: No)	0.809	1.357	0.056
	Age	0.162	0.076	0.152*
	Number of children	-0.485	0.227	-0.166*
**BO**	Constant	4.508	2.855	***-***
	CS	0.202	0.054	0.266**
	Resilience	0.064	0.023	0.189**
	STS	0.512	0.041	0.687**
	Gender (ref; Female)	-0.432	0.595	-0.040
	Education level (ref: BSc)			
	Diploma	-0.193	0.663	-0.017
	Technical institute of nursing	0.684	1.259	0.030
	Postgraduate	0.172	1.102	0.009
	Workplace (ref: Emergency)			
	In-patient	-1.474	0.885	-0.138
	Out-patient	-1.926	0.966	-0.172*
	Marital Status (ref: Married)			
	Single	-0.536	0.954	-0.044
	Divorced/Widowed	-1.138	1.052	-0.061
	Having Children (ref: No)	-1.724	0.953	-0.158
	Age	0.108	0.054	0.133*
	Number of children	0.039	0.163	0.017
**STS**	Constant	15.246	3.779	***-***
	CS	-0.257	0.075	-0.252**
	BO	0.974	0.077	0.726**
	Resilience	-0.072	0.032	-0.159*
	Gender (ref; Female)	0.948	0.818	0.066
	Education level (ref: BSc)			
	Diploma	1.373	.908	0.088
	Technical institute of nursing	0.740	1.736	0.024
	Postgraduate	-0.280	1.519	-0.011
	Workplace (ref: Emergency)			
	In-patient	1.833	1.222	0.128
	Out-patient	3.128	1.325	0.208*
	Marital Status (ref: Married)			
	Single	-0.307	1.316	-0.019
	Divorced/Widowed	0.573	1.455	0.023
	Having Children (ref: No)	-0.017	1.328	-0.001
	Age	-0.185	.074	-0.170*
	Number of children	0.029	.225	0.010

CS: *F* = 11.877, *P* = 0.000, *R*^2^ = 0.51, Adjusted *R*^2^ = 0.467

BO: *F* = 15.314, *P* = 0.000, *R*^2^ = 0.573, Adjusted *R*^2^ = 0.535

STS: *F* = 13.868, *P* = 0.000, *R*^2^ = 0.548, Adjusted *R*^2^ = 0.509

^*^P < *0 .05*

^**^P < *0.001*

The results indicate that STS, CS, and resilience were significant predictors of BO, with higher levels of STS, CS, and resilience associated with higher levels of BO (β = 0.687, *p* < 0.001), (β = 0.266, *p* < 0.001), and (β = 0.189, *p* < 0.001), respectively. Additionally, workplace and age were also significant predictors of BO. Specifically, working in the out-patient department was associated with lower levels of BO compared to working in the emergency department (*p* < 0.05), while older age was associated with higher levels of BO (*p* < 0.05). Overall, the regression model had a good fit as indicated by the significant *F*-value (*F* = 15.314, *p* < 0.001), *R*-squared value (*R*^2^ = 0.573), and adjusted *R*-squared value (*R*^2^_adj = 0.535).

The results of the multiple regression analysis indicate that five predictors had significant effects on STS. Specifically, higher levels of CS were associated with lower levels of STS (β = -0.252, *p* < 0.01), while higher levels of BO were associated with higher levels of STS (β = 0.726, *p* < 0.01). Similarly, higher levels of resilience were associated with lower levels of STS (β = -0.159, *p* < 0.05), and working in an out-patient workplace was associated with higher levels of STS compared to working in an emergency department (β = 0.208, *p* < 0.05). In addition, older age was associated with lower levels of STS (β = -0.170, *p* < 0.05). On the other hand, the other predictors, including Gender, Education level, Marital status, Having Children, and Number of children, had non-significant effects on STS. The regression model had a good fit, as indicated by the significant *F*-value (*F* = 13.868, *p* < 0.001), *R*-squared value (*R*^2^ = 0.548), and adjusted *R*-squared value (*R*^2^_adj = 0.509).

## Discussion

This study is unique in that it explores the correlation between psychological resilience and ProQOL in mental health nurses in the Eastern Region of Saudi Arabia, as well as how sociodemographic factors influence this relationship. Furthermore, the study aimed to identify predictors of compassion satisfaction, burnout, and secondary traumatic stress through multiple regression analysis. The findings revealed that higher levels of resilience were linked to greater compassion satisfaction and fewer symptoms of STS. Furthermore, higher BO scores were found to predict greater compassion satisfaction. Age and the number of children had weak but positive and negative associations with compassion satisfaction, respectively. The study also found that STS, compassion satisfaction, and resilience were significant predictors of BO, with workplace and age also having significant effects. The regression models demonstrated good fits, suggesting that resilience, BO, STS, workplace, and age are important predictors of compassion satisfaction and STS in healthcare professionals.

The study found that the hypothesis of a negative association between burnout and psychological resilience was supported, meaning that nurses with higher levels of resilience experience lower levels of burnout. Additionally, there was a positive correlation between psychological resilience and compassion satisfaction, suggesting that nurses with higher levels of resilience experience higher levels of compassion satisfaction. While there is limited research on this topic specifically among mental health nurses, these findings are consistent with previous research conducted among nurses in general [18, 45, 46]. As a result, it is believed that strategies aimed at improving psychological resilience among nurses will reduce burnout by enhancing CS.

The study found that the mean psychological resilience score for mental health nurses was high, which is consistent with previous studies conducted among nurses in Brazil [47], mental health nurses in the United States [24], and among oncology nurses in Turkey [48]. However, other studies have reported that nurses have moderate [49] or poor [25, 50]. levels of resilience. It is important to note that a high level of resilience is crucial for working in occupations with high levels of stress, such as nursing, as it helps to avoid emotional and physical exhaustion [51]. Additionally, resilience enables individuals to assess stressful situations and use coping strategies more effectively [52].

The present study found that mental health nurses had a high mean score for total psychological resilience, particularly in the hardiness and coping domains. This finding is consistent with previous studies [53, 54], and it may be attributed to the fact that the sample consisted of nurses working in different departments, including inpatient, emergency, and outpatient psychiatry. However, a qualitative study conducted in Palestine suggested that the cultural and religious backgrounds of mental health nurses had a significant impact on their psychological resilience [55]. Hence, resilience may differ based on diverse experiences, cultures, and spiritual values, as evidenced by studies conducted in various countries.

Regarding burnout, the current study revealed that a quarter of mental health nurses had an average level of burnout. This may be due to the numerous challenges mental health nurses face, such as caring for individuals with mental disorders who are at risk of suicide, violence, and other dangers, working in restrictive and isolated settings, dealing with fear and guilt, and maintaining communication with patients and their caregivers while expending tremendous emotional effort [3, 8]. Furthermore, mental health nurses often face increased stress due to acute patients and heavy workloads in mental health facilities [56]. Burnout has been linked to both medical and mental health issues, including but not limited to insomnia, migraines, impaired concentration, chronic tiredness, and irritability. Moreover, burnout can lead to a deterioration in the quality of care and patient satisfaction, as well as an increase in medical errors, malpractice claims, morbidity, and mortality rates [57].

In this study, the ProQOL of mental health nurses was assessed, and it was found that more than half of the nurses had an average level of compassion satisfaction domain, while more than one-third had a good level and only 1.7% had a low level of compassion satisfaction. Regarding the secondary trauma stress domain, about two-thirds of the nurses had an average level, while more than one-quarter had a low level and only 4.5% had a good level. According to a review of the literature, nurses working in psychiatric units tend to have low levels of compassion satisfaction, moderate levels of burnout, and high levels of compassion fatigue. The review suggested that these nurses cope with the challenging work environment by demonstrating self-sacrifice and patience, which can increase their compassion satisfaction. However, the added effort required to work with a vulnerable patient group may also contribute to higher levels of burnout [39]. Thus, it could be argued that high levels of compassion satisfaction experienced by nurses might help to reduce the level of compassion fatigue.

The findings of this study confirm that nurses with stronger psychological resilience experience higher levels of CS, although the number of STS symptoms is negatively associated with it. Previous research conducted among nurses has shown similar relationships between psychological resilience and the ProQOL CS domain [18, 45, 46]. Therefore, it is suggested that strategies aimed at improving psychological resilience among nurses will reduce burnout by enhancing CS. A study conducted in Turkey among 100 psychiatric nurses revealed a positive correlation between CS and resilience, as well as CS and burnout [18].

This study also found that nurses who have children have weak but positive and negative associations with CS, respectively. Although no study has explored the relationship between the ProQOL of mental health nurses and having children, some studies have found that nurses’ demographic characteristics have no significant relationship with CS [58, 59], while others have found that gender, education, managerial position, and experience are linked to CS and CF. A study of the predictors of professional quality of life among 374 nurses in the Philippines found that salary, duration of working duty, and working environment were significant predictors [60]. In addition, a study among mental health nurses in Saudi Arabia [61] and psychiatrists in Egypt [62] reported that married mental health nurses reported higher levels of burnout than single ones. The results of the current study suggest that the presence of children may provide motivation for nurses to bear the difficulties of work. Future studies should focus on investigating the effects of marital status and having children on the ProQOL of healthcare professionals, particularly nurses, using longitudinal and qualitative study designs.

The current study found that age was a predictor of Compassion Satisfaction (CS) and Secondary Traumatic Stress (STS), which is consistent with another study [60]. However, several other studies have reported that age is not significantly related to ProQOL domains [18, 58, 59]. It has been suggested that younger nurses may be new to the institution environment, work policies, professional support, and even workplace leadership and management style, and may therefore experience more stress and burnout [63]. In contrast, older nurses may have better coping strategies and more self-sufficiency, leading to less STS and burnout [60, 64]. Therefore, working with older nurses and their supervision of younger nurses may help reduce stress and burnout among the latter group. However, given the mixed findings in previous studies, longitudinal investigations of nurses’ experiences regarding ProQOL are highly recommended.

Moreover, our study found that nurses working in outpatient settings experienced higher levels of STS compared to those working in emergency departments. However, these findings contradict a previous Australian study that found a correlation between workplace and burnout [65]. The results of our study can be explained by the significant impact of social support on STS, which has been noted in previous research. Studies have shown that nurses with reduced social support are more likely to experience symptoms of STS, particularly when they lack support from colleagues in the workplace [66–68]. Furthermore, our findings are consistent with a cross-sectional study of Greek mental health nurses, which demonstrated that emotional support from colleagues was the only workplace resource that could mitigate the effects of STS and burnout [69].

The current study also found that STS, CS, and resilience were predictors of burnout, which is consistent with previous research [11, 18, 70]. A study conducted in the USA found that stress associated with work due to dealing with traumatized clients was significantly associated with burnout and Compassion Fatigue (CF) [71]. Furthermore, nurses working in clinical units with various difficulties and stressors may ignore their stress symptoms and emotional needs, leading to CF [72]. A narrative review investigating the factors leading to CF among mental health nurses found that the only significant cause was the caseload or number of contacts with traumatized patients [73]. Therefore, interventions to reduce burnout among mental health nurses are vital and may include strong nurse leadership, competency-based education, a positive institutional culture, and self-care strategies [74, 75].

A meta-analysis study confirmed that CF had a strong positive relationship with burnout, while CS had an inverse association with burnout. Negative affect and stress were reported to enhance CF, while positive affect and good social support may enhance CS [58]. Future studies are needed to identify the roles of negative affect and STS among different healthcare providers working with psychiatric patients, and to investigate the synergistic effects of multiple CF factors among large datasets.

Therefore, the results of this study, combined with previous research, emphasize the need to validate the concepts of CF and burnout for the welfare of nurses and to conduct further research related to nurse well-being. Self-care strategies such as self-hypnosis, durable social and peer support, and constructive affirmation have been shown to improve nurses’ capacity for resilience and reduce CF and burnout [20, 76, 77]. Investments in programs that can reduce CF and burnout may also reduce high nurse turnover rates and improve patient care quality. Promising recommendations include workload assignment, mentoring programs, ongoing training, and organizational cultures based on supportive flourishing [78].

## Limitation

The study has some limitations that need to be considered while interpreting the results. Firstly, the study’s cross-sectional design restricts the ability to draw conclusions about causality or changes over time. Secondly, the study was conducted only in the Mental Health Center located in Riyadh, Saudi Arabia, which may restrict the applicability of the results to other populations or settings. Additionally, the study relied on convenience sampling, which may cause sampling bias and influence the representativeness of the sample. Moreover, self-reported measures were used in the study, which may be influenced by response bias and social desirability bias. Lastly, the study’s focus was only on mental health nurses, which may hinder the findings’ comprehensiveness as it did not include other healthcare professionals or patients.

## Conclusion

According to the result of this study we confirm that mental health nurses who exhibit higher levels of psychological resilience tend to experience greater satisfaction in their work with patients. Conversely, those who experience burnout or secondary traumatic stress are likely to have lower levels of resilience. Most of the nurses surveyed had high levels of resilience and average levels of compassion satisfaction, though two-thirds reported average levels of burnout or STS. Nurses with higher resilience tended to have higher levels of compassion satisfaction and lower levels of STS, while those with higher burnout reported greater satisfaction in their work. To improve the professional quality of life of healthcare employees, organizations should consider factors such as resilience, burnout, workplace stress, age, and family responsibilities when developing interventions. Regular assessments and educational programs can help promote a healthy work environment and improve the psychological wellbeing of nurses.

## Data Availability

Data available with the corresponding author upon rational request.

## Abbreviations

ProQOL: Professional Quality of Life
BO: Burnout
STS: Secondary Traumatic Stress
CS: Compassion satisfaction
CD-RISC-25: Connor-Davidson Resilience Scale
IRB: Institutional Review Board
SD: Standard Deviation
Max: Maximum
Min: Minimum
